# Photonic Artifacts in Ratiometric Luminescence Nanothermometry

**DOI:** 10.1021/acs.nanolett.3c01602

**Published:** 2023-07-14

**Authors:** Sander J. W. Vonk, Thomas P. van Swieten, Ario Cocina, Freddy T. Rabouw

**Affiliations:** † Debye Institute for Nanomaterials Science, Utrecht University, Princetonplein 1, 3584 CC Utrecht, The Netherlands; ‡ Optical Materials Engineering Laboratory, ETH Zürich, Leonhardstrasse 21, 8092 Zürich, Switzerland

**Keywords:** photonics, density of optical states, temperature sensing, nanocrystals, lanthanide emission

## Abstract

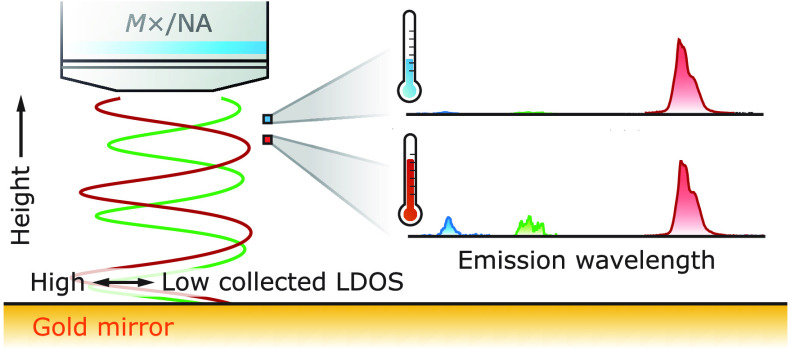

Ongoing developments in science and technology require temperature measurements at increasingly higher spatial resolutions. Nanocrystals with temperature-sensitive luminescence are a popular thermometer for these applications offering high precision and remote read-out. Here, we demonstrate that ratiometric luminescence thermometry experiments may suffer from systematic errors in nanostructured environments. We place lanthanide-based luminescent nanothermometers at controlled distances of up to 600 nm from a Au surface. Although this geometry supports no absorption or scattering resonances, distortion of the emission spectra of the thermometers due to the modified density of optical states results in temperature read-out errors of up to 250 K. Our simple analytical model explains the effects of thermometer emission frequencies, experimental equipment, and sample properties on the magnitude of the errors. We discuss the relevance of our findings in several experimental scenarios. Such errors do not always occur, but they are expected in measurements near reflecting interfaces or scattering objects.

The emission of nanocrystals is often sensitive to temperature, which makes them ideal as remote “nanothermometers”.^1−4^ Recent applications in physics, chemistry, and biology exploit this to measure heat generation and thermal diffusion, for instance, during laser absorption in nanoplasmonics or exposure of living cells to photothermal therapy.^5−7^ In particular, *ratiometric* nanothermometry is popular, in which the local temperature is extracted from the ratio of peak-integrated emission intensities from two electronic transitions of the nanothermometer.^8^ The most common experimental procedure involves (1) calibration of the temperature-dependent emission, (2) embedding of the thermometer nanocrystals in/on a sample of interest, and (3) conversion of recorded emission into temperature.^9−11^ However, the community has recently come to realize that the recorded emission spectrum may be distorted by wavelength-dependent transmission by the sample or wavelength-dependent self-absorption by the thermometer material.^11−13^ These effects are not intrinsic nanothermometer properties and cannot be considered in the external calibration of the emission.

More generally, the effect of the local density of optical states (LDOS) on ratiometric thermometry has not yet been investigated.^14^ This is somewhat surprising, as the entire field of nanophotonics revolves around modulating light–matter interaction using the LDOS. For instance, photonic structures that guide, reflect, or scatter light—by shaping the LDOS at the position of an emitter—steer emission in certain directions.^15−17^ Many sensing methods, including surface-enhanced Raman scattering and cavity-based immunoassays, rely on optical resonances to improve the signal-to-noise ratio and measure small amounts of analyte molecules.^18−20^ Placing an emitter or scatterer on a resonant photonic structure also modifies its output spectrum. This modification is intentional in some cases, such as for the suppression of phonon sidebands in the nitrogen-vacancy emission of diamond, but can also be undesired.^21,22^ One should expect any inhomogeneous optical environment, i.e., a sample containing materials with different refractive indices, to feature a wavelength- and position-dependent LDOS that affects the emission from an embedded nanothermometer. Indeed, our recent temperature-sensing experiments on a microelectronic heater have shown temperature read-out errors of more than 10 K.^23^ The potential impact of an inhomogeneous optical environment on sensing is often however neglected.^12,24−28^

This Letter studies ratiometric luminescent nanothermometers in different optical environments and quantifies photonic artifacts in the temperature read-out. In contrast to our previous work,^23^ we now use samples with known photonic properties and externally controlled heating to investigate the magnitude of photonic errors more generally. Specifically, we placed a monolayer of Er^3+^- or Ho^3+^-doped nanocrystals at controlled distances from a planar Au mirror. The mirror creates a model geometry in which we can systematically vary the LDOS experienced by the nanothermometers. The spatially dependent LDOS describes the change in spontaneous-emission rate and direction due to interference of photon states reflected by the mirror, analogous to the results in Drexhage’s seminal work on fluorescent molecules in front of a mirror.^15^ There is no absorbing material between the nanothermometers and the detector, but the emission spectra are nevertheless distorted and depend strongly on the distance from the Au. This leads to errors in the temperature read-out of up to 100 K using the Er^3+^-based thermometer and up to 250 K using the Ho^3+^-based thermometer, at a constant set temperature of 373 K. These errors are 1 to 2 orders of magnitude larger than those found in our previous work,^23^ because we now use a submonolayer nanocrystal film so that each nanocrystal in a measurement experiences the same precisely defined LDOS. A simple self-interference model reproduces the experiment and explains the difference in read-out error between Er^3+^ and Ho^3+^ from the energy separation of their emission lines. The model further shows how the magnitude of the errors depends on the thermometer sensitivity, *S*_r_, and the numerical aperture NA of the microscope objective. Finally, we conclude with a discussion of experimental environments in which photonic artifacts may or may not be expected. We find that dielectric microspheres—placed on a monolayer of Er^3+^-doped nanothermometers—introduce read-out errors of up to 10 K, which showcases that photonic artifacts may occur even for purely dielectric samples. Our results can guide the optimized selection of a nanothermometer and the experimental setup to minimize photonic artifacts in ratiometric luminescence nanothermometry.

We prepared lanthanide-doped colloidal nanocrystals with temperature-sensitive emission as thermally and photostable nanothermometers. The nanocrystals consist of NaYF_4_, in which a fraction of the Y^3+^ ions are substituted either by a combination of 2% Er^3+^ and 18% Yb^3+^ or by 13.1% Ho^3+^ (Supporting Information Section S1 and Figure S1^9,33^). Fluoride-based materials are a popular choice because of their low vibrational energy, resulting in slow nonradiative relaxation and high luminescence quantum yields.^33^ The temperature-dependent emission of both Er^3+^ and Ho^3+^ has previously been demonstrated. In the Er^3+^-doped nanocrystals, the luminescence is generated via upconversion, where two Yb^3+^ ions transfer 980 nm excitations to a nearby Er^3+^ ion that subsequently emits in the visible (Figure 1a). The emission spectra of the nanocrystals show green emissions centered at 520 and 540 nm, which are due to radiative decay from the ^2^H_11/2_ and ^4^S_3/2_ states (Figure 1b). Er^3+^ acts as a Boltzmann thermometer: thermal equilibrium of the population of the ^2^H_11/2_ and ^4^S_3/2_ states sets in on time scales much faster than spontaneous emission due to rapid energy exchange with lattice vibrations. This makes the luminescence intensity ratio (LIR) of the these states a useful measure for temperature.^9,10^ We excite the Ho^3+^-doped nanocrystals with 445 nm light to obtain emission from the ^5^F_3_ → ^5^I_8_ (blue emission), ^5^F_4_, ^5^S_2_ → ^5^I_8_ (green emission), and ^5^F_3_ → ^5^I_7_ + ^5^F_5_ → ^5^I_8_ (red emission) transitions (Figure 1c,d). The LIR between green emission from the ^5^S_2_ state and red emission from the ^5^F_5_, and ^5^F_3_ states is determined by competition between multiphonon relaxation and cross-relaxation.^34^ We calibrate the thermal response of the Er^3+^ and Ho^3+^ luminescence in a homogeneous optical environment using an external temperature controller (Figure S2).

**Figure 1:**
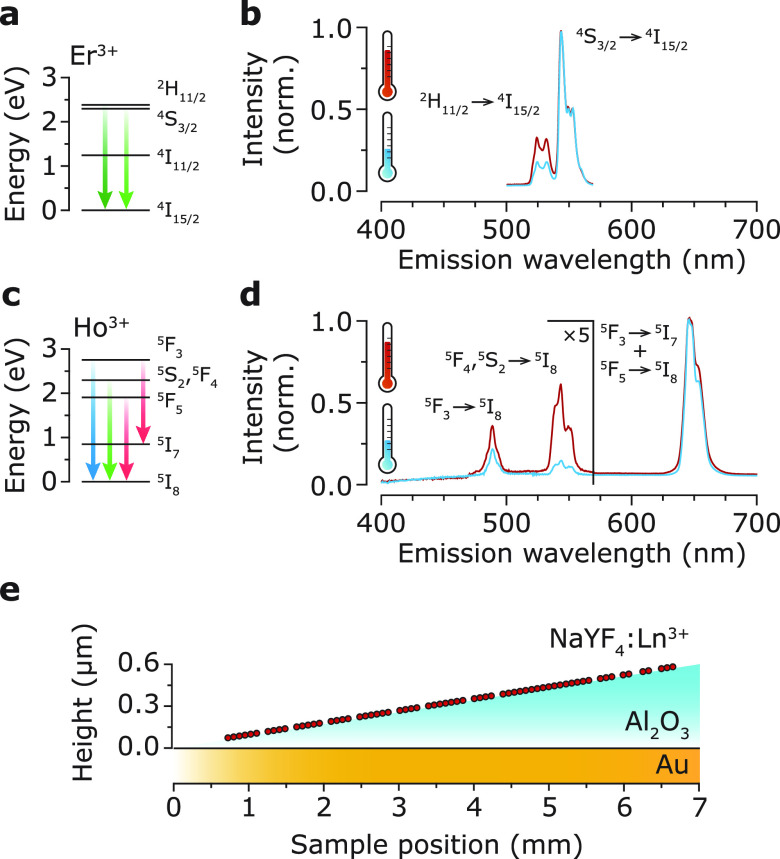
(a) Energy level diagram of Er^3+^ with the relevant emissions as colored arrows. (b) Luminescence spectrum of the NaYF_4_:Er^3+^(2%),Yb^3+^(18%) nanocrystals excited at 980 nm and detected through a bandpass filter transmitting 500–570 nm in a homogeneous sample environment consisting of a film of nanocrystals embedded between PMMA and a glass coverslip with roughly equal refractive indices. At elevated temperature, the relative intensity of the ^2^H_11/2_ → ^4^I_15/2_ emission increases. (c) Same as in (a) but for Ho^3+^. (d) Same experimental procedure as in (b) but for the NaYF_4_:Ho^3+^(13.1%) nanocrystals excited at 445 nm. The blue and green emission lines are magnified by a factor 5. At elevated temperature, the red-to-green ratio, i.e., the ratio between the emission from the ^5^F_3_ → ^5^I_7_ + ^5^F_5_ → ^5^I_8_ (red) and ^5^F_4_, ^5^S_2_ → ^5^I_8_ (green) transitions, decreases. (e) Schematic illustration of the substrate with a controlled photonic environment. The ramped alumina (Al_2_O_3_) spacer contains the lanthanide-doped nanocrystals at submonolayer coverage (Supporting Information Figure S3) and provides a systematically varying distance between the Au mirror and the nanocrystals.

We deposited the lanthanide-doped nanocrystals on a ramped-reflector substrate consisting of a Au mirror covered by an alumina spacer (Figures 1e and S3). The alumina spacer has a thickness increasing from 0 to 600 nm over a distance of 7 mm and is covered with a monolayer of nanocrystals.^35^ An air objective (NA = 0.75; 40×) was used to focus the excitation light and collect the luminescence of the nanocrystals at several locations on the substrate, corresponding to 10–15 nm steps in emitter–mirror distance. The excitation density at fixed laser power oscillates with emitter–mirror distance due to constructive/destructive interference (Figure S4). In our experiments, we minimize the total signal to avoid influence of the excitation rate on our measurements. The shallow ramp angle of the spacer ensures a uniform thickness within 0.1 nm (not including random surface roughness and fabrication imperfections) within the excitation spot. The sample was loaded on a heating stage for measurements at controlled elevated temperatures.

Figure 2a,b compares the Er^3+^ emission spectra recorded from nanothermometers at room temperature and placed at two distances from the Au mirror. Clearly, the spectra are different from each other and from the room-temperature emission spectrum of nanothermometers in a homogeneous environment (dashed lines; reproduced from Figure 1b). The luminescence intensity ratio ^2^H_11/2_/^4^S_3/2_ is higher at *d* = 310 nm (Figure 2a) resembling a temperature higher than room temperature and lower at *d* = 450 nm (Figure 2b) resembling a lower temperature. Laser-induced heating is negligible (Figure S5).

**Figure 2:**
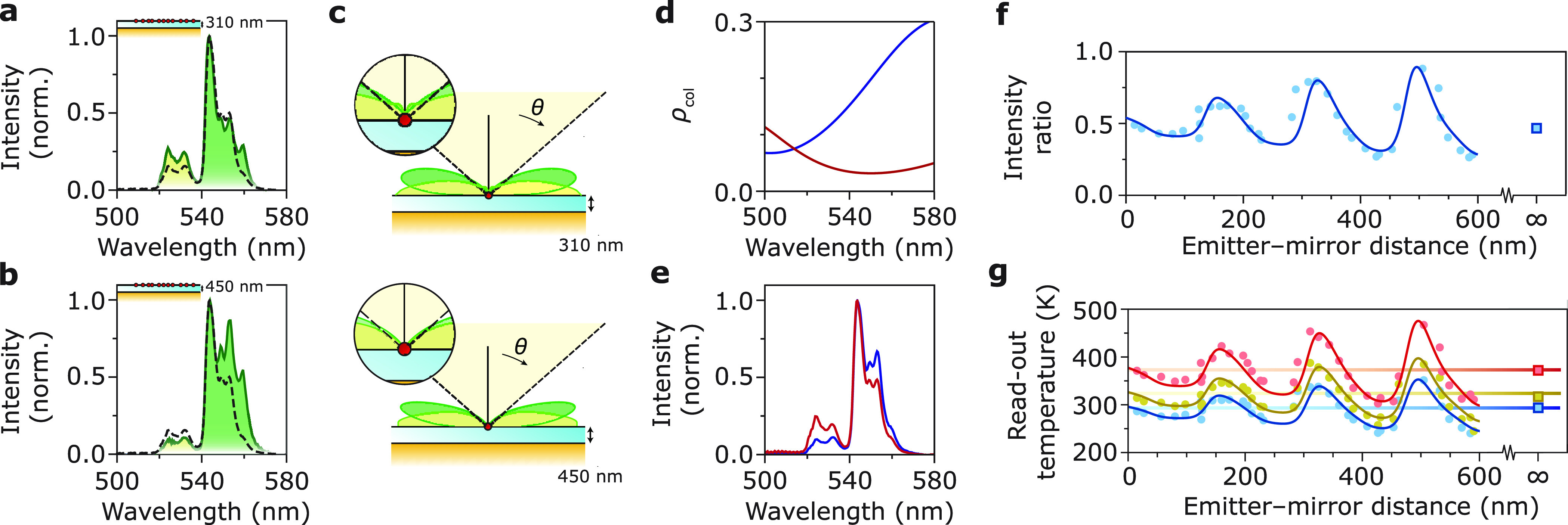
(a) Luminescence of the Er^3+^-doped nanocrystals at an emitter–mirror distance of *d* = 310 nm, recorded using focused 980 nm excitation. The dashed line is the reference spectrum recorded from a homogeneous environment, consisting of a film of nanocrystals embedded between PMMA and a glass coverslip with roughly equal refractive indices. (b) Same as in (a) but for *d* = 450 nm. Both spectra have been normalized to the peak counts to highlight differences in the shape and LIR. (c) Calculated radiation patterns dρ_col_/dθ of emissions at 520 nm (light green) and 540 nm (dark green), for *d* = 310 nm (top) and *d* = 450 nm (bottom) presented as a polar plot.^29^ The objective collects the fraction of the pattern that is within the NA (yellow cone). The geometry has cylindrical symmetry, so the radiation pattern does not depend on azimuthal angle ϕ. (d) Collected LDOS ρ_col_ as a function of wavelength for emitter–mirror separations of 310 nm (red) and 450 nm (blue). (e) Calculated recorded emission spectra at an emitter–mirror separation of 310 nm (red) and 450 nm (blue) following eq 2, using the emission spectrum in a homogeneous environment at room temperature and the wavelength-dependent collected LDOS from panel c. The calculated emission spectra show the same distortions as the measured emission spectra from panel a (compared to red) and panel b (compared to blue). (f) Blue dots: experimental ^2^H_11/2_/^4^S_3/2_ intensity ratios extracted from emission spectra at various *d* values. The ^2^H_11/2_ is integrated between 519–537 nm, and the ^4^S_3/2_ emission is integrated between 537–544 nm. By choosing an integration limit of 544 nm, we exclude emission from the ^2^H_9/2_ from our analysis.^30−32^ Solid lines: intensity ratio as a function of *d* calculated using the collected LDOS averaged over the emission lines (Supporting Information eq S11, Sections S3 and S4). Blue square at *d* = *∞*: intensity ratio in a homogeneous environment. (g) Experimental read-out temperatures of the Er^3+^-doped nanocrystals as a function of *d* measured at set temperatures of 298 K (blue dots), 323 K (yellow dots), and 373 K (red dots). The colored wiggly lines are the read-out temperatures calculated using the collected LDOS and the calibration based on the Boltzmann model, and the colored horizontal lines are the set temperatures.

The distortions in the recorded spectra can be understood in terms of the LDOS at the location of the nanothermometers. We model the Er^3+^ ions as sources of isotropic electric-dipole emission in a planar-mirror geometry with an alumina spacer of variable thickness *d* (Section S4).^36−39^ In the nanocrystals of this study, the excited-state dynamics that populate the emitting states are much faster than radiative decay, which makes the relative populations of these states almost independent of the optical environment.^10,34^ Hence, the photon-emission rate into direction Ω is directly proportional to the differential LDOS dρ/dΩ—i.e., the density of states propagating into direction Ω—at the photon energy. We define the density of photon modes at angles within the collection angles of our microscope as the collected LDOS:
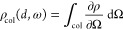
1Figure 2c presents theoretical emission patterns dρ_col_/dθ for the green emissions of Er^3+^. The patterns overlap differently with the range of collection angles of our microscope objective (yellow cone). We predict the recorded luminescence spectrum *I*(*d*, ω) at distance *d* from the Au mirror by scaling the emission spectrum of emitters in a homogeneous environment *I*(*∞*, ω), with the calculated collected LDOS:
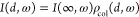
2This expression holds only approximately if the LDOS affects feeding of the emitting levels (Sections S3 and S4).

Figure 2d shows the collected LDOS ρ_col_ as a function of wavelength for the two emitter–mirror distances in Figure 2a,b. This strongly wavelength-dependent ρ_col_ explains why the experimental spectra of Figure 2a,b are distorted. Indeed, using eq 2, we can reproduce the differences between the experimental spectra (Figure 2e). The experimental emission at 560 nm, which is not reproduced by our calculation, is due to third-order upconversion to the ^2^H_9/2_ level as observed previously.^30−32^ We discard this emission from our analysis by choosing an appropriate integration range. Figure 2f shows the experimental intensity ratios between the two green Er^3+^ emission lines as a function of the emitter–mirror distance. The intensity ratio oscillates with *d* and the amplitude increases as a function of *d*. Qualitatively, this is explained by the collected LDOS at the individual emission energies that oscillate with slightly different periodicities, producing a beating wave of the recorded intensity ratio as a function of *d*. The discrepancies between experiment and model are likely due to uncertainties in sample geometry, such as air voids in the layer of nanocrystals, uncertainties in the grain structure of the Au mirror,^39^ or the local thickness and stoichiometry of the Al_*x*_O_*y*_ spacer. We convert the ratios to read-out temperatures using our calibration based on the Boltzmann model (Figures 2g and S2a). Read-out errors are as large as 50 K when the substrate is at room temperature. Heating the substrate to 373 K increases the errors to up to 100 K because of the reduced sensitivity of Boltzmann thermometers at elevated temperatures (eq S34). The calculated intensity ratios based on eq 2 (solid line in Figure 2f) match the experimental ratios. Converting them to expected read-out temperatures using the calibration (solid lines in Figure 2g) reproduces the experimental temperature errors. Our analysis thus demonstrates that in a sample without absorbing medium between nanothermometers and detector, the recorded emission spectrum can still be distorted, translating into significant errors in temperature read-out.

To investigate the impact of the individual emission energies on the read-out errors in ratiometric thermometry, we measure the luminescence of Ho^3+^-doped nanocrystals on the ramped reflector. Compared to Er^3+^, the energy difference between the emission lines of Ho^3+^ is larger by a factor seven. Figure 3a,b shows that the larger difference in emission energies causes a stronger modification of the emission spectrum. The radiation patterns dρ_col_/dθ presented in Figure 3c further illustrate how the completely different collection of the green and red emissions distorts the spectrum. Consequently, the experimental red-to-green intensity ratios heavily oscillate with emitter–mirror distance (Figure 3d; note the logarithmic *y*-scale). Translating the ratios to temperatures produces read-out errors of more than 250 K (Figure 3e). Remarkably, read-out is impossible at the minima of the oscillations because here the recorded intensity ratio is lower than possible in a homogeneous environment at any temperature. Conveniently, the ^2^H_11/2_/^4^S_3/2_ intensity ratio of Er^3+^ and the red-to-green ratio of Ho^3+^ have a similar sensitivity to temperature.^34^ This allows us to identify the energy of the two emission lines as one of the key parameters that determines the photonic distortions.

**Figure 3:**
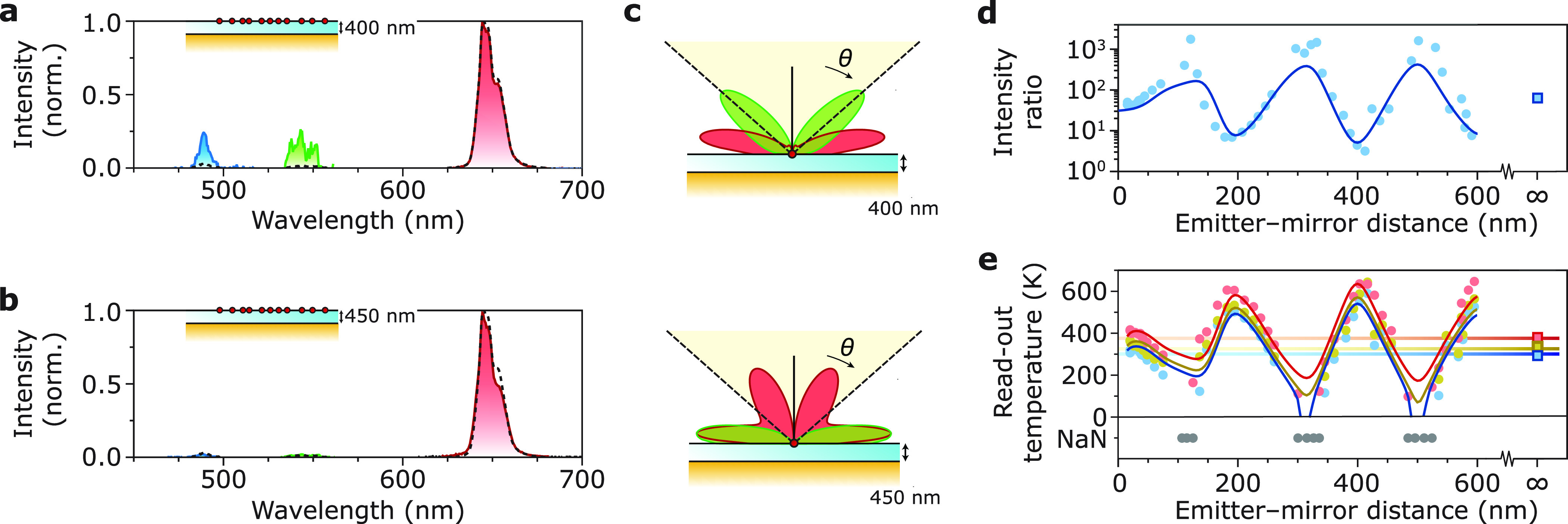
(a) Luminescence of the Ho^3+^-doped nanocrystals at an emitter–mirror distance of *d* = 400 nm, recorded using focused 445 nm excitation. (b) Same as in (a) but for *d* = 450 nm. The dashed line is the reference spectrum recorded from a homogeneous environment, consisting of a film of nanocrystals embedded between PMMA and a glass coverslip with roughly equal refractive indices. Both spectra have been normalized to the peak counts to highlight differences in shape and LIR. (c) Calculated radiation patterns of emissions at 535 nm (green) and 650 nm (red) for *d* = 400 nm (top) and *d* = 450 nm (bottom). The objective collects the fraction of the pattern that is within the NA (yellow area). (d) Blue dots: experimental red-to-green intensity ratios, integrated between 632–670 nm and between 532–560 nm, as a function of *d*. The integration ranges are indicated in (a). Solid lines: intensity ratio as a function of *d* calculated using the collected LDOS averaged over the emission lines (eq S12; Sections S3, and S4). Blue square at *d* = *∞*: intensity ratio in homogeneous environment. (e) Experimental read-out temperatures of the Ho^3+^-doped nanocrystals for various emitter–mirror distances measured at set temperatures of 298 K (blue dots), 323 K (yellow dots), and 373 K (red dots). Solid lines: read-out temperatures calculated using the collected LDOS and the shell model for a Ho^3+^ concentration of 13.1%.

What is the relevance of our experiments for ratiometric luminescence (nano)thermometry in applications? The large errors we found, in excess of 10% in absolute temperature, would be unacceptable for most applications. Luckily, smaller errors may be expected for dielectric or biological samples with lower reflectivities.

To predict the magnitude of photonic distortions in various sample environments, we set up a simplified model for a reflective surface of arbitrary reflectivity *R* (Section S5.1). Considering a hypothetical experimental setup collecting all emission from a thermometer particle at a distance *d* from the interface, the ratio of the collected LDOS at two emission lines with frequency difference Δω and average frequency ω̅ is
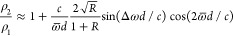
3where *c* is the speed of light in the medium where the nanothermometer particles are embedded. As a function of *d* the ratio behaves as a beating wave with envelope periodicity 2π*c*/Δω and carrier-wave periodicity π*c*/ω̅ . The carrier-wave periodicity is clearly visible in the measurements on the Er^3+^-based (π*c*/ω̅ = 165 nm; Figure 2f) and Ho^3+^-based (π*c*/ω̅ = 185 nm; Figure 3d) nanothermometers. Equation 3 can be adapted to account for a maximum collection angle of the optics that collect the luminescence (eq S18). The maximum errors in temperature read-out near an interface Δ*T*_max_ are given by the extreme values of ρ_2_/ρ_1_ (eqs S21 and S33):
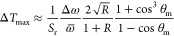
4

They depend on the intrinsic nanothermometer properties *S*_r_ and Δω/ω̅, reflectivity *R* of the sample environment, and the maximum collection angle θ_m_ of the experimental setup.

Figure 4a shows a good match between the experimental maximum read-out errors Δ*T*_max_ for our ramped-reflector measurements (squares) to the calculated values based on eq 4 (solid lines). For the calculations, we used the numerical aperture (NA = 0.75) of our setup and *R* = 1.

**Figure 4:**
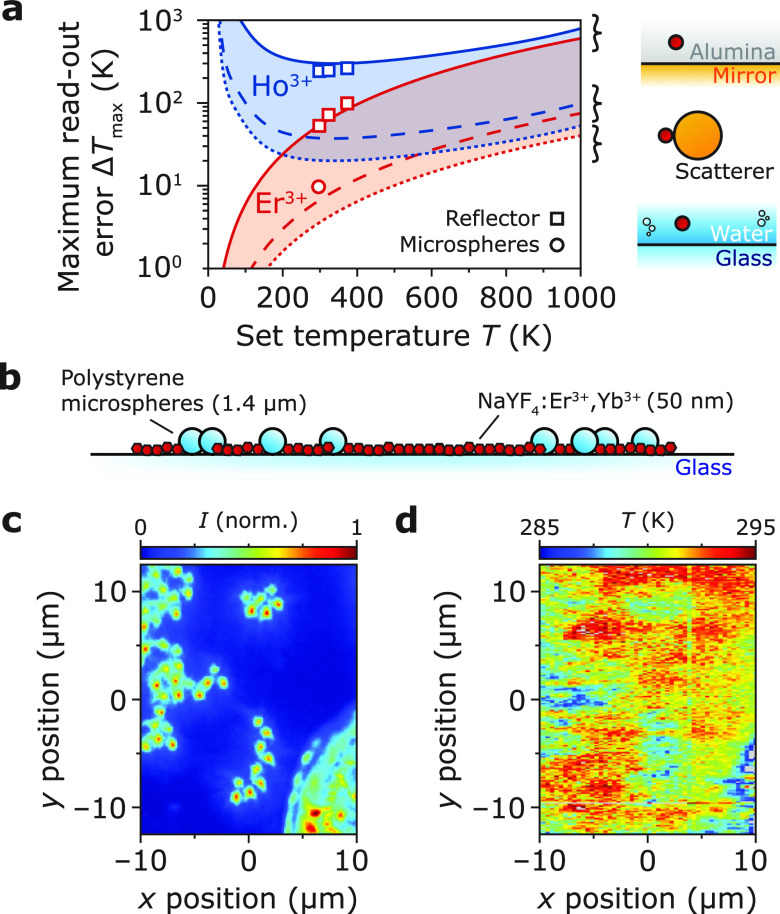
Photonic artifacts in different environments. (a) Maximum read-out errors of the Er^3+^- and Ho^3+^-doped thermometers located in alumina near a perfect reflector (thin solid lines), on the surface of a strongly polarizable particle (thick dashed lines), and in water near a slab of glass (thin dashed lines) for an NA of 0.75. The squares are the experimental maximum read-out errors near a reflector for the three different set temperatures (Figures 2g and 3e) and the circle in a purely dielectric sample at room temperature (Figure 4d), which are defined as the maximum deviation of the experimental read-out temperature from the set temperature. Δ*T*_max_ depends on the set temperature through *S*_r_.(b) Schematic sample geometry of a purely dielectric sample consisting of polystyrene microspheres (1.4 μm diameter) and NaYF_4_:Er^3+^,Yb^3+^ nanoparticles on a glass substrate. (c) Dark-field microscopy image of the dielectric sample. The high-intensity spots correspond to locations of polystyrene microspheres, which have a large scattering cross section. (d) Two-dimensional temperature map of the same area as in (c) at room temperature. The read-out temperature is approximately 10 K lower at the location of the polystyrene microspheres.

The dotted lines in Figure 4a estimate the maximum read-out temperature in biological *in vitro* experiments. For this estimate, we consider the effect of a weakly reflecting water–glass interface (*R* = 0.004). Even for this weakly reflective interface, calculated errors of up to tens of kelvin remain. *In vitro* experiments may further be complicated by the inhomogeneous structure of cells containing various organelles with different optical properties. For example, mitochondria have a higher refractive index (*n* = 1.41) and nuclei a lower refractive index (*n* = 1.34–1.35) than the cytoplasm (*n* = 1.35–1.37).^40,41^ Our experiments and calculations suggest that such refractive index contrasts could introduce photonic errors in ratiometric thermometry experiments on living cells. As one example, we highlight the surprising intracellular temperature observed in ref (42), which are difficult to unify with the existing understanding of heat generation and dissipation in cells.^43^ For the experimental conditions in ref (42), eq 4 estimates potential photonic artifacts of up to Δ*T*_max_ = 11 K (see Section S5.4 for the calculation). The potential artifacts thus have the same estimated order of magnitude as the surprising intracellular temperature variations observed in ref (42) and may be important for the interpretation of the observations.

In addition to reflective surfaces, samples may contain particles with dimensions on the nano- to micrometer scale,^44^ which scatter light. We also constructed a simple model for the effect of a nearby scattering particle on a temperature reading (Section S5.2). The predicted temperature errors are included in Figure 4a as dashed lines. To test the occurrence of photonic artifacts due to scattering, we prepared a purely dielectric sample (Figure 4b) consisting of polarizable polystyrene (PS) microspheres (1.4 μm, refractive index *n* = 1.6^45^) and Er^3+^-doped nanoparticles (*n* = 1.48) on a glass substrate (*n* = 1.5). Dark-field microscopy reveals the locations of polystyrene microspheres on the sample (Figure 4c). Recording a temperature map on the same sample area at room temperature (Figure 4d) shows spatial variations of the read-out temperature exceeding the expected variations due to noise. From the negative correlation between the scattering intensity *I*_scat_ in Figure 4c and the read-out temperature in Figure 4d (Figure S8) we conclude that the vicinity of PS microspheres corrupts temperature measurements and leads to lower read-out temperatures compared to the set temperature. The read-out errors match our simple model for scattering particles approximately (circle in Figure 4). While a more quantitative calculation of the expected distortions in this complex geometry is too challenging,^45,46^ this experiment showcases the issue of photonic artifacts even for purely dielectric samples.

Our experiments and calculations above suggest a significant problem for ratiometric luminescence thermometry in any inhomogeneous sample environment. This suggestion is however inconsistent with our previous experiments near a reflective heating spiral,^23^ where the photonic errors were noticeable but 1 to 2 orders of magnitude smaller than those found in the current work. We wrote previously that the photonic distortions were “expected to be subtle as the ^2^H_11/2_ and ^4^S_3/2_ emissions have nearly the same wavelength”. We now show that the distortions can produce temperature errors in excess of 50 K, even for Er^3+^ at room temperature (Figure 4), while previously we estimated them at 4 K.^23^ The much smaller temperature errors in our previous work were likely the result of the thick layer of nanothermometers used. By recording the emission from a thick layer, we previously averaged the photonic distortions over multiple oscillations (Figures 2f and 3d). Similarly, many *in vivo* applications of luminescence thermometry investigate temperature gradients on length scales much larger than micrometers, which would sufficiently average out potential photonic distortions, so that they are no longer an issue. However, the strategy of averaging negatively affects the spatial resolution of luminescence nanothermometry, which is exactly one of its attractive features.

If high spatial resolutions are desired and volume averaging is not an option, eq 4 provides guidelines on how to minimize photonic errors in ratiometric nanothermometry. Thermometers with low Δω/ω values are preferable. Infrared-emitting thermometers with small ω̅, which are preferred in biological systems because of the large penetration depth of near-infrared light,^12,13,47^ may thus not be the ideal choice when it comes to minimizing photonic artifacts. Our model predicts lower read-out errors when the numerical aperture of the experimental setup is large (large θ_m_). Careful selection of the thermometer and the experimental equipment could thus minimize the distortions that are inherently induced by reflective interfaces.

One could also quantify the frequency-dependent collected LDOS inside the sample environment—for instance with a reference measurement at room temperature as we did in our previous work^23^—and use this to correct the recorded spectrum in postanalysis. However, this solution would work only for static samples. Moreover, various relevant samples, including *in vitro* biological and biomedical experiments, may not be easily temperature-controlled for a proper reference measurement. A strategy applicable to situations in which reference measurements are difficult or impossible is the acquisition of real-time information on the LDOS at the position of the thermometer. Lin et al.^19^ previously demonstrated this for plasmon-enhanced Raman spectroscopy. Applied to luminescence thermometry, this would require embedding a reference emitter with a broad and temperature-insensitive emission spectrum into the sample, whose spectrum would provide real-time information on the LDOS during the experiment.^44^

Finally, we emphasize here that a more qualitative interpretation of luminescence nanothermometry is possible, even if absolute recorded temperatures contain systematic photonic errors. For example, characterizing spatial variations or time dynamics in the temperature could provide useful qualitative insights in heat generation and flow. It is, however, important to consider the potential contributions of spatial and temporal variations in the photonic properties of a sample. These could be the cause of the apparent temperature gradients or time dynamics in part or even entirely.

## Supporting Information Available

Experimental methods, details of calibration/analysis procedures, derivation of simple analytical models to predict magnitude of photonic artifacts (PDF)
