# Supplementary material for: Photonic Artifacts in Ratiometric Luminescence Nanothermometry
Source: Nano Lett. 2023 Jul 14;23(14):6560–6. doi: 10.1021/acs.nanolett.3c01602 (PMC10375589; doi:10.1021/acs.nanolett.3c01602)
Supplement: Supplementary file 1 — nl3c01602_si_001.pdf [file nl3c01602_si_001.pdf]

Supporting Information for

Photonic artifacts in ratiometric luminescence nanothermometry

Sander J.W. Vonk [1], *Thomas P. van Swieten* [1], *Ario Cocina* [2], *and Freddy T. Rabouw* [1*]

[1]Debye Institute for Nanomaterials Science, Utrecht University, Princetonplein 1, 3584 CC Utrecht, The Netherlands

[2]Optical Materials Engineering Laboratory, ETH Zürich, Leonhardstrasse 21, 8092 Zürich, Switzerland

[*] Corresponding Author: f.t.rabouw@uu.nl

S1 Methods

S1.1 Preparation of the substrate

A four-inch Si wafer was diced into 1×1 cm^2 chips. Each chip was cleaned by ultrasonication for 2 minutes in acetone and in isopropylalcohol, and blown dry with N_2. Then, a layer of 280-nm-thick positive electron-beam resist (Allresist, CSAR AR-P 6200.09) was spincoated on the chip. Reference marks were etched using electron-beam lithography. After electron-beam exposure (Vistec Lithography, EBPG 5200+), the resist was developed (Allresist, AR 600-546) for 1 minute. The marks were etched into Si using a HBr-based inductively-coupled-plasma reactive-ion etching procedure. The resist was removed by exposing the surface to an oxygen plasma (PVA TePla, GIGAbatch 310M) at 600 W for 5 minutes. Finally, the chip was cleaned in a piranha solution, consisting of a 1:1 mix of sulfuric acid (Sigma-Aldrich, 95.0–97.0%) and hydrogen peroxide (VWR Chemicals, 30%), for 15 minutes.

The silicon substrates were loaded into a thermal evaporator (Kurt J. Lesker, Nano 36). Approximately 200 nm of Au (Umicore pellets) was evaporated onto the substrate at a rate of 1 nm s^{-1}. The sample consisting of a Si substrate coated with Au layer was then transferred to the vacuum chamber of a magnetron sputterer (PVD75, Kurt Lesker) and mounted on a four-inch rotating holder for deposition of Al_2O_3. The evaporator chamber was evacuated to a pressure below 8×10^{-6} Torr. A custom-built metallic shadow mask was then mounted on a non-rotating support at close distance from the substrate, partially covering the sample from the sputtered material. Al_2O_3 was deposited via reaction of Al sputtered from an Al target (2.00 inch diameter × 0.125 inch thick, 99.999 % purity, Kurt Lesker) and partial O_2 injection in addition to Ar during the deposition. The volumetric flow ratio O_2/Ar was kept to 20%, which gave an overall Al_2O_3 evaporation rate of 7 nm min^{-1}. By rotating the holder with respect to the fixed shadow mask during deposition, the area of the sample covered by the mask was gradually decreased such to expose fresh Au areas to the material flux. The sample holder was rotated during the deposition every 8 minutes by 0.6 degrees for 14 times.

The lanthanide-doped nanocrystals were synthesized using the procedure of Geitenbeek *et al.*[S1]. A monolayer of nanocrystals was spincoated on the ramped reflector from a diluted dispersion (concentration 5 mg mL^{-1}) to obtain the substrate that was used for further spectroscopic experiments. For the purely dielectric sample, we spincoated 50 μL of an equal-volume mixture of an aqueous dispersion of polystyrene microspheres (1.39 ± 0.04 μm, microParticles GmbH) and a dispersion of Er^{3+}-doped nanocrystals onto a #1.5 glass coverslip.

S1.2 Spectroscopic measurement

All measurements were performed using a home-built optical setup based on a Nikon Ti-U inverted microscope body.

Ramped reflector For the upconversion $NaYF_4$:Er^{3+},Yb^{3+} nanocrystals, a 980-nm laser (OBIS LX 980 nm) was guided to the sample by a 50/50 beamsplitter (Thorlabs, BSW26R) and focused by a 40× Nikon CFI Plan Fluor (NA = 0.75) air objective, resulting in an excitation intensity on the sample of approximately 1 kW cm^{-2}. The $^2H_{11/2}$,$^4S_{3/2}$ $\rightarrow$$^4I_{15/2}$ emission lines were selected using a band-pass filter (Chroma, ET535/70M). For the $NaYF_4$:Ho^{3+} nanocrystals, a 445-nm laser (OBIS LX 445 nm FC) operated at approximately 25 W cm^{-2} was guided to the sample by a long-pass dichroic (Semrock, FF01-468/SP-25) in combination with a short-pass filter (Semrock, FF01-468/SP-25) to reject the long-wavelength tail of the laser line, resulting in an excitation intensity on the sample of approximately 25 W cm^{-2}. A long-pass filter (Semrock, BLP01-458R-25) was used to block the reflected excitation light. All lanthanide emission was collected by the same objective and collimated outside of the microscope using a relay lens system and subsequently focused on a mechanical slit (200 μm) at the entrance of an Andor Kymera 193i spectrometer and dispersed with a 150 lines/mm grating blazed at 500 nm on an Andor iXon EMCCD. All emission spectra were corrected for the wavelength-dependent collection and detection efficiency using an Ocean Optics HL-3 calibration lamp. We adjust the excitation power for

each measurement to compensate for interference of the excitation laser above the mirror and prevent effects of laser heating on the read-out temperature.

Microsphere sample We correlated the distortions of the read-out temperature with the presence of polystyrene microspheres by first imaging the polystyrene particles using dark-field microscopy. Here, the sample was illuminated by an oil-immersion objective (Nikon CFI Plan Apochromat Lambda 100×, NA = 1.45) under normal incidence using a 400-μm pinhole in the Fourier plane of the white-light lamp (Lumencor SOLA). In the emission path, specular reflections were blocked using a mask in the Fourier plane. The scattered light was mostly unaffected by the Fourier mask. The resulting image on our EMCCD contrasts the strongly scattering polystyrene particles from the weakly scattering thermometer particles. After that, a two-dimensional temperature map was constructed of the same area as the dark-field microscopy image employing wide-field illumination by the 980-nm laser and a 150 mm defocusing lens. We placed a 3.5-mm aperture in a relayed Fourier plane of the objective to match the numerical aperture of the air objective used for the ramped-reflector measurements. The erbium luminescence was projected onto the entrance slit (150 μm) of our spectrometer and dispersed with the 150 lines/mm grating. This gives a two-dimensional intensity map on our EMCCD detector where the horizontal position gives the emission wavelength and the vertical position the position along the slit (y-direction). From this measurement, a one-dimensional temperature map was constructed by integrating the H and S emission for every y-position and subsequently converted to temperature using our calibration curve. Finally, a two-dimensional temperature map was obtained by scanning our sample over the slit (x-direction, orthogonal to y-direction) for a total range of 20 μm in steps of 0.4 μm using a 3-axis Piezo sample stage (Mad City Labs, Nano-LPS).

S2 Characterization and calibration

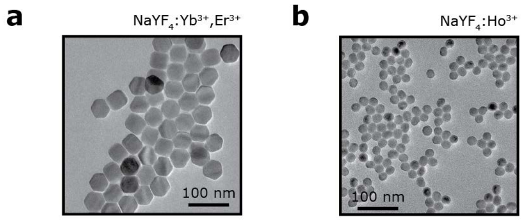

Figure S1 | Characterization of lanthanide-doped nanocrystals. (a) Electron-microscopy image of $NaYF_4$:Er^{3+},Yb^{3+} nanoparticles. The particles have a diameter of 46.3 ± 3.3 nm (mean ± standard deviation). Previous studies on similar nanocrystals have shown a quantum yield for the visible $^2H_{11/2}$,$^4S_{3/2} \rightarrow {}^5I_{15/2}$ emission of $\eta \approx 15\%$ upon resonant excitation[S2] or $\eta \approx 0.1\%$ for upconversion excitation.[S3] **(b)** Same as **a**, but for Ho^{3+}-doped nanocrystals with a diameter of 22.4 ± 1.8 nm (mean ± standard deviation). We estimate the efficiencies upon resonant excitation of $\eta \approx 1\%$ for the $^5F_3 \rightarrow {}^5I_8$ emission, $\eta \approx 1\%$ for the $^5S_2$,$^5F_4 \rightarrow {}^5I_8$ emission, and $\eta \approx 10\%$ for the $^5F_3 \rightarrow {}^5I_7$ and $^4F_5 \rightarrow {}^5I_8$ emissions, based on a comparison of the photoluminescence decay rates of our 13.1%-doped nanocrystals with those of microcrystalline $NaY_{0.75}Gd_{0.25}F_4$ doped with 0.5% Ho^{3+} from Ref. [S4]. The electron microscopy images were acquired with a Tecnai 20 transmission electron microscope.

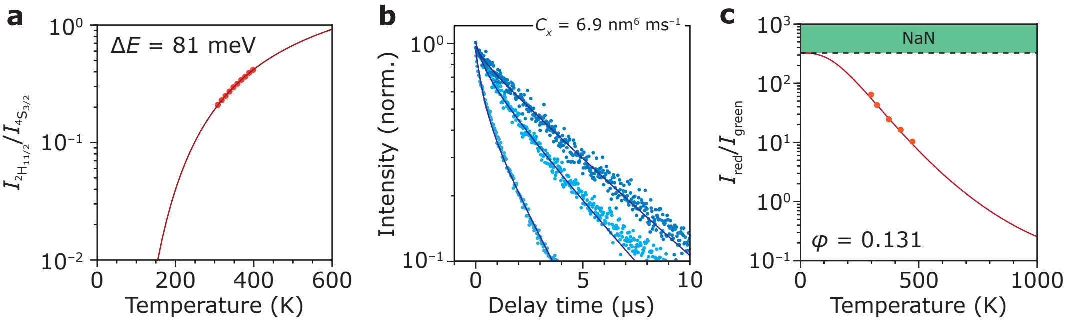

Figure S2 | Calibration of the luminescence thermometers. **(a)** Intensity ratio of $^2H_{11/2}$ and $^4S_{3/2}$ emission lines (red dots) of the $NaYF_4$,Yb^{3+},Er^{3+} nanocrystals in a homogeneous optical environment (film of nanocrystals embedded between PMMA and glass coverslip) for set temperatures between 30 and 120°C. We fit the data points using a Boltzmann model (red solid line) accounting for the thermal population of the $^2H_{11/2}$ energy level

$$Y_{\mathrm{BM}} = \frac{g^{^2\mathrm{H}_{11/2}}}{g^{^4\mathrm{S}_{3/2}}} \frac{k_{\mathrm{r}}^{^2\mathrm{H}_{11/2}}}{k_{\mathrm{r}}^{^4\mathrm{S}_{3/2}}} \mathrm{e}^{-\Delta E/k_{\mathrm{B}}T}, \tag{1}$$

where T is the temperature, $\Delta E = 81$ meV is the energy separation between the two levels, k_{r}^i is the spontaneous emission rate of state i, and g^i is the degeneracy of level i. We use the fitted line to convert the measured intensity ratio Y on the ramped reflector to apparent temperatures T' in Figure 2g of the main text. **(b)** Photoluminescence decay curves of the blue energy level (445 nm excitation) of Ho^{3+}-doped microcrystalline $NaYF_4$. The Ho^{3+} concentration varies from 0, 5, and 12 % (dark to light blue). We use a global-fitting procedure based on a microscopic cross-relaxation model and find a cross-relaxation constant $C_{\mathrm{x}} = 6.9\ \mathrm{nm}^6\mathrm{ms}^{-1}$. **(c)** Same as **a**, but for the Ho^{3+}-doped nanocrystals. The solid red line is an analytical calculation of the red-to-green intensity ratio as a function of temperature using the radiative rates, energy-transfer rates, multi-phonon relaxation rates of the energy levels in the visible and a statistical distribution of local-ion environments in $NaYF_4$ (doping concentration 13.1%) as reported in Ref. S4. We assume that cross relaxation is mediated by a Förster-type energy-transfer mechanism. We adapted the cross-relaxation constant of the blue energy level (panel **b**) to account for a slighter higher intensity ratio Y over the entire temperature range for doped $NaY_{1-x}Ho_xF_4$ (this work) versus $NaY_{0.75-x}Gd_{0.25}Ho_xF_4$ (Ref. S4). Again, the solid line is used to convert the intensity ratio Y measured on the ramped reflector to an apparent temperature in Figure 3e of the main text. We observe that intensity ratios $Y > 160$ are unphysical since they exceed the highest possible red-to-green ratio in a homogeneous optical environment posed by the radiative decay rates and the steady-state populations of the emitting levels at zero kelvin.

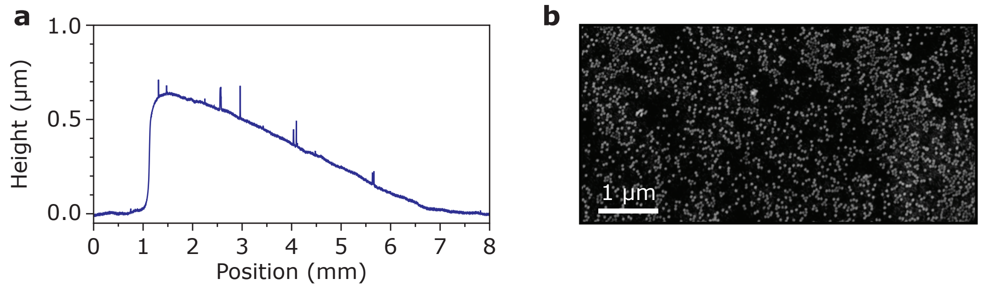

Figure S3 | Characterization of ramped reflector sample. **(a)** Height measurements of the Al_2O_3 ramp on the silicon–Au sample using a stylus profilometer (DektakXT, Bruker). The distance between the starting position of the profile scan and an engraved marking on the sample was measured to determine the spacer thickness for all optical measurements at different positions. **(b)** Scanning electron microscopy image of the ramped reflector coated with Er-based thermometer particles showing submonolayer coverage of the substrate. **(b)** Scanning electron microscopy image of the ramped reflector coated with Er-based thermometer particles showing sub-monolayer coverage.

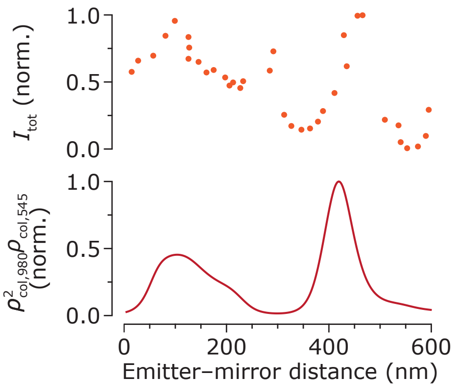

Figure S4 | Modulation of the signal strength (a) Normalized total emission intensity I_{tot} of the Er^{3+}-based thermometer (integrated between 500–570 nm) for varying emitter–mirror distances between 0–600 nm at constant acquisition parameters and constant laser power. Note that data points are not extracted from the same data set presented in Figure 2 of the main text, where we adapted the laser power at every emitter–mirror distance to avoid laser heating and achieve roughly equal signal strength for each measurement. Here, variations in the total emission intensity might be due to differences in nanocrystal coverage, excitation power, and collection efficiency. (b) Theoretical emission intensity as a function of emitter–mirror distance. We model the total emission intensity as the product of excitation efficiency $\rho^2_{col,980}$—which scales with the square of the collected LDOS at the laser wavelength because we rely on second-order photon upconversion—and collection efficiency $\rho_{col,545}$ at the average emission wavelength. We observe that the variations of the experimental total intensities match this theoretical model qualitatively.

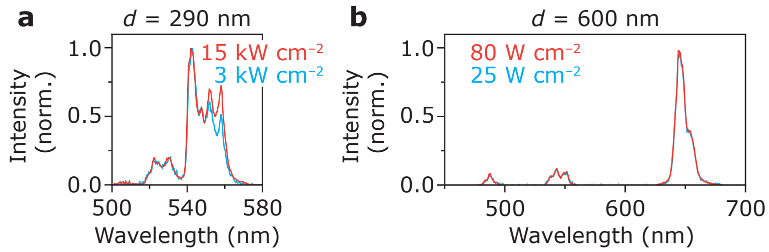

Figure S5 | Check for laser heating. (a) Room-temperature upconversion luminescence of Er^{3+} at an emitter–mirror distance $d = 290$ nm at low excitation power (blue, 3 kW cm^{-2}) and high excitation power (red, 15 kW cm^{-2}). We observe that the relative intensity from the $^2H_{9/2}$ energy level increases with excitation power indicating a higher-order upconversion process populating this energy level. The relative intensity of $^2H_{11/2}$ and $^4S_{3/2}$ emission is constant, indicating that the Au substrate and lanthanide-doped nanocrystals are not heated by the laser. **(b)** Same as **a**, but for Ho^{3+} at low excitation power (blue, 25 W cm^{-2}) and high excitation power (red, 80 W cm^{-2}). Also here, there is no indication of laser heating on the temperature readout. In all measurements on Er^{3+} and Ho^{3+}, we used excitation powers approximately corresponding to those of the spectra measured at low excitation power (blue). However, for each measurement, we minimized the excitation power to achieve sufficient signal within a reasonable time frame, while minimizing higher-order upconversion.

S3 Effects of intrinsic properties and the photonic environment on the luminescence intensity ratio

In this work, we characterize the influence of the photonic environment on the luminescence intensity ratio (LIR) of two emission lines from a luminescence thermometer. We will show that there is a fundamental but subtle difference of the LDOS dependence on the LIR for Boltzmann thermometers and energy-transfer thermometers.

We start by considering a Boltzmann thermometer with energy levels A and B. B is higher in energy than A by an amount ΔE. If the population exchange between the two levels by phonon absorption/emission is much faster than decay from the levels, then their fractional populations $p_{\mathrm{A}}, p_{\mathrm{B}}$ are in a quasi-Boltzmann equilibrium[S5]. We approximate in our work that the Er^{3+}-based thermometer remains a Boltzmann thermometer in all photonic environments since the LDOS does not boost the radiative decay rate to values comparable with (or faster than) the nonradiative coupling rate between the levels. The total photon emission rate $k_{\mathrm{tot},i}$ from level i is then given by

$$k_{\mathrm{tot},i}(\omega_i, T) = p_i(T)\rho_{\mathrm{tot}}(\omega_i)k_{\mathrm{r},i}, \tag{2}$$

where the rate constant for radiative decay in a homogeneous environment $k_{\mathrm{r},i}$ is distorted by the photonic environment via the total LDOS ρ_{tot} (normalized to the LDOS in a homogeneous environment) at the emission frequency ω_i.

In an experiment, not all photon modes can be collected by the microscope objective. The collected photon emission rate

$$k_{\mathrm{col},i}(\omega_i, T) = p_i(T)\rho_{\mathrm{col}}(\omega_i)k_{\mathrm{r},i}. \tag{3}$$

accounts for the experimental collection efficiency $\rho_{\mathrm{col}}/\rho_{\mathrm{tot}}$ at the emission frequency ω_i and is proportional to the recorded signal strength at frequency ω_i. The LIR of a Boltzmann thermometer with two infinitely narrow emission lines equals the ratio of collected photon emission rates of the lines:

$$Y_{\mathrm{BM}}(T) = \frac{k_{\mathrm{col,B}}(\omega_{\mathrm{B}}, T)}{k_{\mathrm{col,A}}(\omega_{\mathrm{A}}, T)} = \frac{g_{\mathrm{B}}k_{\mathrm{r,B}}}{g_{\mathrm{A}}k_{\mathrm{r,A}}}\frac{\rho_{\mathrm{col}}(\omega_{\mathrm{B}})}{\rho_{\mathrm{col}}(\omega_{\mathrm{A}})}\mathrm{e}^{-\Delta E/k_{\mathrm{B}}T}, \tag{4}$$

which depends only on ρ_{col} at the two emission frequencies, not on ρ_{tot}. We observe that the LIR depends on *internal* emitter properties such as the vacuum radiative rates $k_{\mathrm{r},i}$ and degeneracies g_i of the two levels and the energy separation ΔE between the two levels, and on *external* parameters such as temperature and the collected LDOS ratio of the emission frequencies ω_{A} and ω_{B}. In a homogeneous environment—for example nanocrystals in vacuum or dispersed in a solvent—the ratio of collected LDOS $\rho_{\mathrm{col}}\omega_{\mathrm{B}})/\rho_{\mathrm{col}}(\omega_{\mathrm{A}})$ is equal to unity because the collected photon-emission rates of the two levels depend in the same way on the surrounding medium and collection optics. This means we effectively calibrate the internal properties of the emitter when placing the nanocrystals in a homogeneous environment. For nanocrystals placed in an inhomogeneous environment, a common misconception is that only these internal properties determine the LIR, but this is not the case. The collected LDOS in an inhomogeneous environment can be different for the two emission lines, $\rho_{\mathrm{col}}(\omega_{\mathrm{B}}) \neq \rho_{\mathrm{col}}(\omega_{\mathrm{A}})$, which can lead to significant distortions of the LIR which is converted into read-out errors of the local temperature.

Next, we consider an energy-transfer thermometer with energy levels A and B where the populations are fed from some higher energy level on a time scale comparable to radiative decay from A and B. Here, the population of the energy levels is, in general, determined by a competition between temperature-dependent nonradiative processes, temperature-independent nonradiative processes, and LDOS-dependent radiative processes. Following the same derivation as for the Boltzmann thermometer, but with LDOS-dependent fractional populations of the energy levels, we obtain a general expression for the LIR from a thermometer with infinitely narrow emission lines:

$$Y_{\mathrm{ET}}(T) = \frac{p_{\mathrm{B}}[\rho_{\mathrm{tot}}(\omega_{\mathrm{B}})k_{\mathrm{r,B}}; T]}{p_{\mathrm{A}}[\rho_{\mathrm{tot}}(\omega_{\mathrm{A}})k_{\mathrm{r,A}}; \rho_{\mathrm{tot}}(\omega_{\mathrm{B}})k_{\mathrm{r,B}}; T]}\frac{k_{\mathrm{r,B}}}{k_{\mathrm{r,A}}}\frac{\rho_{\mathrm{col}}(\omega_{\mathrm{B}})}{\rho_{\mathrm{col}}(\omega_{\mathrm{A}})}. \tag{5}$$

We describe here that the populations of the levels depend on the total LDOS at the emission frequencies ω_A and ω_B and the LIR, additionally, on the collected LDOS. As we discuss below (Section S4), radiative decay processes are slow compared to nonradiative processes in our Ho^{3+}-doped nanocrystals, so the population ratio p_B/p_A is almost independent of the LDOS. In this limit, the dependence on total LDOS drops out and we can approximate that

$$Y_{\mathrm{ET}}(T) \approx \frac{p_{\mathrm{B}}(T)}{p_{\mathrm{A}}(T)} \frac{k_{\mathrm{r,B}}}{k_{\mathrm{r,A}}} \frac{\rho_{\mathrm{col}}(\omega_{\mathrm{B}})}{\rho_{\mathrm{col}}(\omega_{\mathrm{A}})}. \tag{6}$$

Also here, placing the energy-transfer thermometer in a homogeneous environment calibrates the intrinsic radiative and nonradiative rates of the emitter.

S4 Modeling the intensity ratio from a self-interference model

To calculate the luminescence intensity ratio of the lanthanide-doped nanoparticles in our experiments, we model our nanoparticles as emitters of isotropic electric-dipole emission embedded in a four-layer mirror geometry (Figure 1e of the main text). This is a good model for the luminescence of lanthanide ions, which constitutes a combination of transitions between crystal-field components with different dipole orientations.[S6] All optical transitions of Er^{3+} and Ho^{3+} studied in this work are electric-dipole transitions[S7]. In our model, layer 1 is the central layer containing the emitters with constant thickness h, layer 2 is the top semi-infinite layer with dielectric constant ϵ_2, layer 3 is the layer below the emitter with variable thickness d and dielectric constant ϵ_3, and layer 4 is the semi-infinite bottom layer. We assume a fixed distance between the emitters and the top layer $s_{12} = h$ such that the emitters are located on the interface between layers 1 and 3 and the separation between the emitters and interface 3/4 is varied with the thickness d of the spacer layer 3. In this geometry, the polarization-averaged LDOS for an electric-dipole transition is given by:[S8]

$$\frac{\rho}{\rho_0} = \int_0^{u_{\mathrm{m}}} \mathrm{Im}\Bigg\{ \Bigg[\frac{(1+r_{\mathrm{s}}^{12}\mathrm{e}^{-2k_0\sqrt{\epsilon_1}l_1 s_{12}})(1+r_{\mathrm{s}}^{134})}{1-r_{\mathrm{s}}^{12}r_{\mathrm{s}}^{134}\mathrm{e}^{-2k_0\sqrt{\epsilon_1}l_1 s_{12}}} + \frac{(1+r_{\mathrm{p}}^{12}\mathrm{e}^{-2k_0\sqrt{\epsilon_1}l_1 s_{12}})(1+r_{\mathrm{p}}^{134}) - 2u^2(r_{\mathrm{p}}^{12}\mathrm{e}^{-2k_0\sqrt{\epsilon_1}l_1 s_{12}} + r_{\mathrm{p}}^{134})}{1-r_{\mathrm{p}}^{12}r_{\mathrm{p}}^{134}\mathrm{e}^{-2k_0\sqrt{\epsilon_1}l_1 s_{12}}} \Bigg] \frac{u}{l_1} \Bigg\} \mathrm{d}u, \tag{7}$$

where ρ_0 is the radiative transition rate of the nanocrystal in a homogeneous optical environment with dielectric constant ϵ_1, $k_0 = 2\pi/\lambda$ is the vacuum wave vector, $l_j = -\mathrm{i}\sqrt{\epsilon_j/\epsilon_1 - u^2}$, and $u = k_\parallel/k$ the fractional in-plane momentum of electromagnetic radiation. From this expression we can calculate the total LDOS ρ_{tot} using $u_{\mathrm{m}} = \infty$ and the collected LDOS ρ_{col} using $u_{\mathrm{m}} = \mathrm{NA}/\sqrt{\epsilon_1}$, where NA is the numerical aperture of the microscope objective. The collected LDOS is dependent on the spacer thickness d via the three-layer Fresnel coefficients $r_{\mathrm{s,p}}^{134}$, given by

$$r_{\mathrm{s,p}}^{134} = \frac{r_{\mathrm{s,p}}^{13} + r_{\mathrm{s,p}}^{34}\mathrm{e}^{-2k_0\sqrt{\epsilon_1}l_3 d}}{1 + r_{\mathrm{s,p}}^{13}r_{\mathrm{s,p}}^{34}\mathrm{e}^{-2k_0\sqrt{\epsilon_1}l_3 d}}, \tag{8}$$

where $r_{\mathrm{s,p}}^{13}$ and $r_{\mathrm{s,p}}^{34}$ are the Fresnel coefficients for reflection of s-and p-polarized light on the interface between layers 1/3 and 3/4, respectively. The Fresnel coefficient for the interface between medium i and j is given in terms of the dielectric constants ϵ_i and the emission direction u by:

$$r_{\mathrm{s}}^{ij} = \frac{l_i - l_j}{l_i + l_j}, r_{\mathrm{p}}^{ij} = \frac{\epsilon_j l_i - \epsilon_i l_j}{\epsilon_j l_i + \epsilon_i l_j}. \tag{9}$$

We approximate layer 1, which in the experiment constitutes $NaYF_4$ nanocrystals, organic ligands, and air voids, as a homogeneous layer with $\epsilon_1 = 2.2$ (Ref. S9) with fixed thickness $h = 20$ nm. We place the emitters in our calculation at the top surface of the nanocrystal layer ($s_{12} = 20$ nm). Layer 2 is air with dielectric constant $\epsilon_2 = 1$, layer 3 is an Al_2O_3 spacer with variable thickness d ($\epsilon_3 = 2.78$ [S10]). High-energy excitation at 445 nm generated background fluorescence from Al_2O_3, which we subtracted from the emission spectra in Figure 3 of the main text[S11]. The bottom layer 4 is a reflective Au surface

(wavelength-dependent dielectric constant ϵ_4 obtained from McPeak *et al.*[S12]).

In a homogeneous optical environment, all lanthanide ions in a nanocrystal have the same emission spectrum. Most emission peaks in the spectrum of a lanthanide are due to a set of transitions between the excited-state multiplet and the ground-state multiplet. Both multiplets can be split by the crystal field of the host lattice, which gives rise to the distinct lineshape of lanthanide emission. The splitting of the excited-state multiplet due to the crystal field is typically much smaller than the thermal energy, and thermal population exchange is therefore much faster than radiative decay. The population distribution within a crystal-field-split multiplet is therefore unaffected by the total LDOS. The population distribution between different multiplets relevant for thermometry may or may not depend on LDOS, as discussed above (Section S3).

Er^{3+} used in this work acts as a Boltzmann thermometer and the multiplet populations are independent of LDOS. The emission spectrum $I(d, \omega)$ collected from nanocrystals at a distance d from the reflector is easily calculated from the emission spectrum in a homogeneous environment $I(\infty, \omega)$ and the collected LDOS $\rho_{\mathrm{col}}(d, \omega)$:

$$I(d, \omega) = I(\infty, \omega)\rho_{\mathrm{col}}(d, \omega). \tag{10}$$

We measured the homogeneous emission spectrum $I(\infty, \omega)$ from of a film of nanocrystals embedded between a glass coverslip and PMMA (all refractive indices $n \approx 1.5$). We then calculate the expected intensity ratio Y between the two emission lines of the Er^{3+}-based thermometer (Figure 2f of the main text) as a function of emitter–mirror separation d by integrating eq S10 over the emission lines:

$$Y(d) = \frac{\int_{\omega_{\mathrm{G1}}} I(d, \omega)\mathrm{d}\omega}{\int_{\omega_{\mathrm{G2}}} I(d, \omega)\mathrm{d}\omega}, \tag{11}$$

where ω_{G1} and ω_{G2} denote the range of emission frequencies corresponding to the two transitions.

For Ho^{3+}, eq S10 holds only approximately, because Ho^{3+} is an energy-transfer thermometer and the populations of the multiplets emitting green, and red light depend weakly on ρ_{tot}. We account for this with correction factors C_{R} and C_{G} in the calculation of expected intensity ratio Y from the experimental spectrum $I(\infty, \omega)$ in the homogeneous environment:

$$Y(d) = \frac{C_{\mathrm{R}}}{C_{\mathrm{G}}} \frac{\int_{\omega_{\mathrm{R}}} I(\infty, \omega)\rho_{\mathrm{col}}(d, \omega)\mathrm{d}\omega}{\int_{\omega_{\mathrm{G}}} I(\infty, \omega)\rho_{\mathrm{col}}(d, \omega)\mathrm{d}\omega}. \tag{12}$$

To calculate C_{R} and C_{G}, we use the full model for the excited-state dynamics of the Ho^{3+}-based thermometer described in Ref. S4, but with increased/decreased radiative rates $k_{\mathrm{r},i} \to \rho_{\mathrm{tot}}(\omega_i)k_{\mathrm{r},i}$ depending on the thermometer–reflector separation d. Here ρ_{tot} is calculated at the center emission frequencies of the Ho^{3+} emission lines: $\{2\pi c/\omega_{\mathrm{B}}, 2\pi c/\omega_{\mathrm{G}}, 2\pi c/\omega_{\mathrm{R}}\} = \{485, 540, 650\}$ nm. The altered radiative decay rates change the photon emission yields from the red- and green-emitting levels by an amount C_{R} and C_{G}, respectively. The correction factors depend on d and on the set temperature but always have values close to unity, $C_{\mathrm{R}}/C_{\mathrm{G}} \in [0.85, 1.37]$, while the intensity ratios are distorted by up to over a factor 10. The photonic distortions of Ho^{3+} can therefore still be understood conceptionally from the simple eq S10.

S5 Read-out temperatures in different photonic environments

S5.1 Intensity-ratio distortions for emitters near reflective surfaces

Here, we derive a simplified model for the photonic artifacts in luminescence thermometry to gain insights into the effects of various experimental parameters.

We consider an isotropic electric-dipole source in a homogeneous medium with dielectric constant ϵ_1 with z-coordinate $z = d$ above a reflective 2D interface at $z = 0$ (Figure S6a). By reciprocity, the

electric-field strength of light emitted into direction (θ, ϕ) is proportional to the electric-field strength of a plane wave coming from (θ, ϕ) on the position of the emitter. This is equal to

$$\mathbf{E}_{\mathrm{p,s}} = \mathbf{E}^0_{\mathrm{p,s}} + \mathbf{r}^{\mathrm{p,s}} \odot \mathbf{E}^0_{\mathrm{p,s}} \mathrm{e}^{2ikd\cos\theta}, \tag{13}$$

for s- and p-polarized light, where $k = \sqrt{\epsilon_1} 2\pi/\lambda$. Here, $\mathbf{E}^0_{\mathrm{p,s}}$ are electric-field vectors for s/p-polarized plane waves coming from (θ, ϕ), and $\mathbf{r}_{\mathrm{p,s}}$ takes into account the amplitude and direction of the electric field of the reflected plane wave given by

$$\mathbf{E}^0_{\mathrm{p}} = \begin{pmatrix} \cos\theta\cos\phi \\ \cos\theta\sin\phi \\ \sin\theta \end{pmatrix}, \mathbf{E}^0_{\mathrm{s}} = \begin{pmatrix} -\sin\phi \\ \cos\phi \\ 0 \end{pmatrix}, \mathbf{r}_{\mathrm{p}} = r_{\mathrm{p}} \begin{pmatrix} 1 \\ 1 \\ -1 \end{pmatrix}, \text{ and } \mathbf{r}_{\mathrm{s}} = r_{\mathrm{s}} \begin{pmatrix} 1 \\ 1 \\ 1 \end{pmatrix}. \tag{14}$$

We will make the approximation that the Fresnel coefficients are angle- and polarization-independent, $r_{\mathrm{s}}(\theta) = r_{\mathrm{p}}(\theta) = r$, and purely real. The emission intensity I_{tot} into a direction (θ, ϕ), equal to the incoherent sum of s- and p-polarized light, is given then by

$$I_{\mathrm{tot}}(\theta, \phi) = \frac{1}{2}\Big(|\mathbf{E}_{\mathrm{p}}|^2 + |\mathbf{E}_{\mathrm{s}}|^2\Big) = 1 + R + 2r\cos(2kd\cos\theta)\cos^2\theta, \tag{15}$$

where $R = |r|^2$ is the intensity reflectivity.

We can calculate the maximum collected LDOS $\rho_{\mathrm{col,max}}$, achieved in the limit that the microscope objective collects all light propagating in the positive z-direction, by integrating the emission intensity I_{tot} over all possible emission angles $\theta \in [0, \pi/2]$ and $\phi \in [0, 2\pi]$ into the half-space of the microscope objective

$$\rho_{\mathrm{col,max}} = \frac{1}{4\pi}\int_0^{2\pi}\int_0^{\pi/2} I_{\mathrm{tot}}(\theta, \phi)\sin\theta \mathrm{d}\theta \mathrm{d}\phi \approx \frac{1}{2}\left[1 + R + \frac{r}{kd}\sin(2kd)\right]. \tag{16}$$

where in the second step we have neglected terms of order $(kd)^{-2}$ and higher since these terms do not significantly contribute in our experiments ($kd > 1$ for 540-nm emission and emitter–mirror separations >100 nm). We normalize the collected LDOS by the total solid angle 4π to retrieve the collected LDOS in a homogeneous medium far away from the interface. We observe that the maximum collected LDOS has a constant offset and term that oscillates with emitter–interface separation d at a frequency $2k$. At large kd, the amplitude of the oscillation is small with respect to the constant offset.

We can account for the finite NA of microscope objectives by integrating eq S16 over the range of emission angles $\theta \in [0, \theta_{\mathrm{m}}]$ and $\phi \in [0, 2\pi]$ that can be collected. We obtain a modified expression for the collected LDOS

$$\rho_{\mathrm{col}} \approx \frac{1}{2}\Big\{(1+R)(1-\cos\theta_{\mathrm{m}}) + \frac{r}{kd}\Big[\sin(2kd) - \cos\theta_{\mathrm{m}}\sin(2kd\cos\theta_{\mathrm{m}})\Big]\Big\}, \tag{17}$$

where again we neglected terms of order $(kd)^{-2}$ and higher. We see that ρ_{col} now has two terms oscillates with d at slightly different frequencies $2k$ and $2k\cos\theta_{\mathrm{m}}$. At large kd, the amplitude of the oscillation is still small with respect to the constant offset.
To illustrate the effect of reflectivity R, NA and frequency difference $\Delta\omega$ on the intensity-ratio distortions, we calculate the collected LDOS ratio $\rho_{\mathrm{col},2}/\rho_{\mathrm{col},1}$ between two emission lines at frequencies $\omega_1 = k_1 c$ and $\omega_2 = k_2 c$. We use that, for large values of d, the amplitude of the oscillating terms in ρ_{col} (eq S17) is much smaller than the constant offset. This allows us to expand $\rho^{-1}_{\mathrm{col},1} = [a + f(kd)]^{-1} = a^{-1} + a^{-2}f(kd) + \mathcal{O}\big[f(kd)^2\big]$, where a and $f(kd)$ denote the constant and oscillating terms in $\rho_{\mathrm{col},1}$. We arrive at the expression

$$\frac{\rho_{\mathrm{col},2}}{\rho_{\mathrm{col},1}} \approx 1 + \frac{c}{\overline{\omega}d}\frac{2\sqrt{R}}{1+R}\Big[\frac{\cos(2\overline{\omega}d/c)\sin(\Delta\omega d/c) - \cos^2\theta_{\mathrm{m}}\cos(2\overline{\omega}d\cos\theta_{\mathrm{m}}/c)\sin(\Delta\omega d\cos\theta_{\mathrm{m}}/c)}{1-\cos\theta_{\mathrm{m}}}\Big], \tag{18}$$

using $\sin(2\omega_1 d/c) - \sin(2\omega_2 d/c) = 2\cos(2\overline{\omega} d/c)\sin[(\omega_1 - \omega_2)d/c]$ to express the sum of sines as a beating wave with envelope frequency $\Delta\omega = \omega_1 - \omega_2$ and modulation frequency $2\overline{\omega} \approx \omega_1 + \omega_2$, and similarly for the terms containing $\cos\theta_\mathrm{m}$. For a perfect microscope objective $\theta_\mathrm{m} = \pi/2$ collecting all nanocrystal emission, we obtain

$$\frac{\rho_{\mathrm{col},2}}{\rho_{\mathrm{col},1}} \approx 1 + \frac{c}{\overline{\omega} d}\frac{2\sqrt{R}}{1+R}\big[\cos(2\overline{\omega} d/c)\sin(\Delta\omega d/c)\big], \tag{19}$$

which is eq. 3 of the main text. The function $\rho_{\mathrm{col},2}/\rho_{\mathrm{col},1}$ oscillates rapidly with d within an envelop function that varies more slowly. We obtain an expression for the envelope of $\left[\rho_{\mathrm{col},2}/\rho_{\mathrm{col},1}\right]_{\mathrm{env}}$ by setting the value of the two rapidly oscillating cosine factors at ± 1:

$$\left[\frac{\rho_{\mathrm{col},2}}{\rho_{\mathrm{col},1}}\right]_{\mathrm{env}} \approx 1 \pm \frac{c}{\overline{\omega} d}\frac{2\sqrt{R}}{1+R}\left[\frac{\sin(\Delta\omega d/c) + \cos^2\theta_\mathrm{m}\sin(\Delta\omega d\cos\theta_\mathrm{m}/c)}{1-\cos\theta_\mathrm{m}}\right]. \tag{20}$$

This slowly oscillating function approaches unity for $d \to \infty$, corresponding to an effectively homogeneous optical environment. The extreme point, deviating maximally from unity, is at $d \to 0$. At this point, we define the maximum possible distortion factor $\kappa_\pm$

$$\kappa_\pm = 1 \pm \frac{\Delta\omega}{\overline{\omega}}\frac{2\sqrt{R}}{1+R}\frac{1+\cos^3\theta_\mathrm{m}}{1-\cos\theta_\mathrm{m}}, \tag{21}$$

which depend on the relative frequency difference $\Delta\omega/\overline{\omega}$, the reflectivity R of the interface and the maximum emission angle θ_m that falls within the NA of the microscope objective.

To visualize the effect of the reflectivity on the intensity-ratio distortions of the two emission lines $\rho_{\mathrm{col},2}/\rho_{\mathrm{col},1}$, we calculate the distortions as a function of emitter–interface separation for a mirror–air (Figure S6b, $R = 1$) and a glass–air interface (Figure S6c, $R = 0.04$) using a NA = 0.75 ($\theta_\mathrm{m} = \arcsin \mathrm{NA}$) and for emission lines at $2\pi c/\omega_1 = 520$ nm and $2\pi c/\omega_2 = 540$ nm (approximately the two emission lines of Er^{3+}). We observe that evaluating the full model (blue lines, eq S17) and the simplified model (red lines, eq S18) give approximately the same collected LDOS ratio as a function of emitter–interface separation. Also, the dashed lines in Figure S6b–c show that eq S21 approximately gives the maximum distortions of the intensity ratio.

Perhaps surprisingly, we observe that the maximum distortion factor $\kappa_\pm$ due to a glass–air interface ($R = 0.04$) is as high as 40% of the distortion factor due to a perfect mirror ($R = 1$), while the reflectivity R is 25× smaller. This can be understood from eq S21, which shows a weaker than square-root dependence of the distortion factor on R. This highlights that reliable temperature readout is difficult, even near interfaces that are barely reflective, such as in biological environments. Figure S6d shows the maximum distortion factor $\kappa_\pm$ as a function of the NA = $\sin\theta_\mathrm{m}$ by numerically optimizing the analytical model $\rho_{\mathrm{col},2}/\rho_{\mathrm{col},1}$ (dots, eq S17) and evaluating the approximation (solid lines, eq S21) for emission lines at $2\pi c/\omega_1 = 520$ nm and $2\pi c/\omega_2 = 540$ nm near a mirror–air interface. The distortions diverge for $\theta_\mathrm{m} \to 0$ which shows the importance of maximizing the collection angle θ_m to minimize distortions. In Figure S6e, we investigated the distortions for one emission line at 520 nm and a redshifted emission line 520 nm $+\,\Delta\lambda$ near a mirror–air interface (NA = 0.75) for the analytical model (dots) and the approximation (solid lines). We observe that the maximum distortion factors increase/decrease approximately linearly with the wavelength shift $\Delta\lambda$.

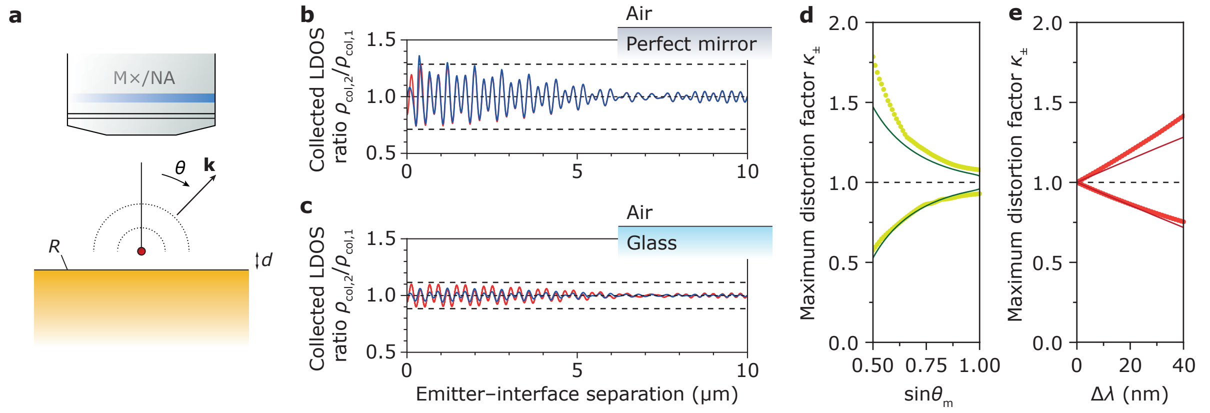

Figure S6 | Intensity-ratio oscillations near an interface. (a) Source of electric-dipole radiation in medium 1 with permittivity ϵ_1 at distance d from an interface with reflectivity R. **(b)** Collected intensity ratio versus emitter–interface separation for two emission lines at 520 nm and 540 nm near a perfect mirror ($R = 1$) for the analytical model (blue line, eq S17) and the approximation (red line, eq S18) for a numerical aperture NA $=$ 0.75. The dashed lines show the maximum distortion factors $\kappa_\pm$ calculated with reflectivity $R = 1$, numerical aperture NA $= 0.75$, and relative frequency difference $\Delta\omega/\omega = 0.038$. **(c)** Same as **b**, but for a glass–air interface ($R = 0.04$). The amplitude of the oscillations is approximately 40% of the amplitude for the perfect mirror. **(d)** Maximum distortion factor $\kappa_\pm$ versus NA $= \sin\theta_\mathrm{m}$ for two emission lines at 520 nm and 540 nm from the analytical model (eq S17, dots) and the approximation (eq S18, solid lines). **(e)** Maximum distortion factor $\kappa_\pm$ for an emission line at 520 nm and a redshifted emission line with wavelength difference $\Delta\lambda$, from numerically optimizing the analytical model (eq S17, dots) and evaluating the approximation (eq S21, solid lines).

S5.2 Apparent temperature for emitters on a polarizable particle

We consider an isotropic emitter in medium 1 with dielectric constant ϵ_1 directly on a polarizable particle with polarizability α and radius a that sits in the origin of the coordinate system (Figure S7a). The emitter is located between the objective and the polarizable particle. By reciprocity, the electric-field strength of the light emitted by the emitter into direction (θ, ϕ) is proportional to the electric-field strength of a plane wave coming from the z-direction on position $\mathbf{r} = a(\sin\theta\cos\phi, \sin\theta\sin\phi, \cos\theta)^\mathrm{T}$. Here, we model the particle as a polarizable dipole source driven by the plane wave. For x- and y-polarized light the total electric field on the position of the emitter is given by

$$\begin{aligned} \mathbf{E}_{(x)} &= \mathbf{E}^0_{(x)} \mathrm{e}^{\mathrm{i}kz} + \overleftrightarrow{\mathbf{G}}_\mathrm{NF} \cdot \left(\alpha \mathbf{E}^0_{(x)} \right) \\ \mathbf{E}_{(y)} &= \mathbf{E}^0_{(y)} \mathrm{e}^{\mathrm{i}kz} + \overleftrightarrow{\mathbf{G}}_\mathrm{NF} \cdot \left(\alpha \mathbf{E}^0_{(y)} \right) \end{aligned} \tag{22}$$

where we use the near-field Green's function

$$\overleftrightarrow{\mathbf{G}}_\mathrm{NF} = \frac{\mathrm{e}^{\mathrm{i}ka}}{4\pi\epsilon_0\epsilon_1 a^3} \left[3\left(\frac{\mathbf{r}\otimes\mathbf{r}}{a^2} \right) - \overleftrightarrow{\mathbf{I}} \right] \tag{23}$$

to calculate the electric-field strength on the position of the emitter in the near field of the polarizable particle. Using reciprocity, we calculate the intensity of isotropic emission into (θ, ϕ) by the incoherent sum of x- and y-polarized light intensities on the position of the emitter. The maximum collected LDOS $\rho_\mathrm{col,max}$ is equal to the total intensity integrated over all emission angles $\theta \in [0, \pi/2]$ and $\phi \in [0, 2\pi]$ into the half-space of the microscope objective:

$$\rho_\mathrm{col,max} = \frac{1}{4\pi} \frac{1}{2|\mathbf{E}_0|^2} \int_0^{2\pi} \int_0^{\pi/2} \left(|\mathbf{E}_{(x)}|^2 + |\mathbf{E}_{(y)}|^2 \right) \sin\theta \, \mathrm{d}\theta \mathrm{d}\phi, \tag{24}$$

which can be evaluated analytically to

$$\rho_{\mathrm{col,max}} = \frac{1}{2}\left\{1+\frac{|\alpha|^2}{8\pi^2\epsilon_0^2\epsilon_1^2a^6}-\mathrm{Re}(\alpha)\left[\frac{6ka-(6+k^2a^2)\sin ka}{4\pi\epsilon_0\epsilon_1k^3a^6}\right]+\mathrm{Im}(\alpha)\left[\frac{2(k^2a^2-3)-(6+k^2a^2)\cos ka}{4\pi\epsilon_0\epsilon_1k^3a^6}\right]\right\}. \tag{25}$$

We use a simple Lorentz oscillator model for the frequency-dependence of polarizability α of a particle with resonance frequency ω_0:

$$\alpha = \frac{\alpha_0}{(\omega^2-\omega_0^2)+\mathrm{i}\gamma\omega}, \tag{26}$$

where γ is the damping rate and α_0 is a prefactor. Figure S7b shows $|\alpha|^2$ for resonance wavelength $2\pi c/\omega_0 = 500$ nm and $\gamma = 2\times10^{14}$ Hz. We consider the limit of strong polarizability $\alpha \gg 4\pi\epsilon_0 R^3$, where the $|\alpha|^2$ term of eq S25 dominates. In this limit, the maximum distortion factor $\kappa = [\rho_{\mathrm{col,max},2}/\rho_{\mathrm{col,max},1}]_{\max}$ can be simplified for two emission lines of the thermometer centered around $\overline{\omega}$, at by $\omega_{1,2} = \overline{\omega}(1\pm\Delta/2)$ with relative frequency difference $\Delta = \Delta\omega/\overline{\omega}$ to

$$\kappa(\overline{\omega}) = \frac{|\alpha[\overline{\omega}(1+\Delta/2)]|^2}{|\alpha[\overline{\omega}(1-\Delta/2)]|^2}. \tag{27}$$

To investigate the dependence of the maximum distortion factor κ on the relative frequency difference Δ, we expand eq S27 around $\Delta = 0$ up to first order

$$\kappa(\overline{\omega}) = 1+\frac{2\overline{\omega}}{|\alpha(\overline{\omega})|}\left(\frac{\mathrm{d}|\alpha|}{\mathrm{d}\omega}\right)_{\omega=\overline{\omega}}\Delta+\mathcal{O}(\Delta^2). \tag{28}$$

For thermometer emission strongly red-detuned from the particle resonance $\overline{\omega} \ll \omega_0$, $|\alpha|^2$ is frequency-independent (Figure S7b) and the maximum distortion factor κ is unity (Figure S7c)

$$\lim_{\overline{\omega}\to 0}\kappa(\overline{\omega}) \approx 1. \tag{29}$$

For two emission lines with average frequency $\overline{\omega}$ very close to the resonance frequency ω_0, we find

$$\lim_{\overline{\omega}\to\omega_0}\kappa(\overline{\omega}) \approx 1-2\frac{\Delta\omega}{\overline{\omega}}, \tag{30}$$

which gives a linearly increasing/decreasing distortion factor κ as a function of the relative frequency difference $\Delta\omega/\overline{\omega}$ (Figure S7c). Lastly, we investigate the regime where the average emission frequency of a thermometer is strongly blue-detuned from the resonance frequency $\overline{\omega} \gg \omega_0$. Here $|\alpha|^2$ scales very strongly with frequency ω^{-4} (Figure S7b). In this limit, we find a distortion factor

$$\lim_{\overline{\omega}\to\infty}\kappa(\overline{\omega}) \approx 1-4\frac{\Delta\omega}{\overline{\omega}}, \tag{31}$$

which also gives a linearly increasing/decreasing distortion factor κ (Figure S7c), but with a larger prefactor compared to the resonant regime.

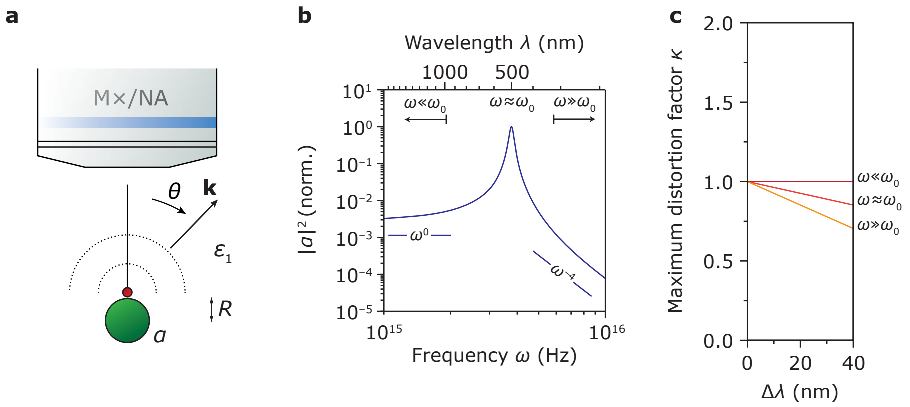

Figure S7 | Intensity-ratio distortions on a polarizable particle. (a) We consider an isotropic emitter in medium 1 (ϵ_1) on a polarizable particle with polarizability α and radius R. **(b)** $|\alpha|^2$ from a simple Lorentz oscillator model with resonance wavelength $2\pi c/\omega_0 = 500$ nm and damping rate γ of 2×10^{14} Hz. For low emission frequencies with respect to the resonance frequency $\omega \ll \omega_0$, $|\alpha|^2$ is frequency independent and photonic artifacts on the emission spectrum of the thermometer of emitter are absent. For high emission frequencies compared to the resonance frequency $\omega \gg \omega_0$, $|\alpha|^2$ decreases rapidly with frequency ω^{-4}. **(c)** Maximum distortion factor κ of the luminescence intensity ratio between one emission line at 520 nm and one redshifted emission line at 520 nm + $\Delta\lambda$ far red-detuned from the particle resonance (dark red line, eq S29), at the resonance frequency (red line, eq S30), and blue-detuned emission frequencies from the particle resonance (orange line, eq S31).

S5.3 Temperature errors for an arbitrary intensity-ratio near a reflective surface

We can calculate the apparent temperature T' near a reflector at temperature T with absolute distortion on the intensity ratio $Y(\kappa_\pm - 1)$. To first order, the apparent temperature T' is given by

$$T' \approx T + Y(\kappa_\pm - 1)\frac{\mathrm{d}T}{\mathrm{d}Y}. \tag{32}$$

Using the definition of relative sensitivity $S_\mathrm{r} = Y^{-1}\mathrm{d}Y/\mathrm{d}T$, which is a commonly accepted parameter to characterize thermometer performance, and filling in eq S21, we obtain

$$\Delta T \approx \left(\frac{\Delta\omega}{\overline{\omega}}\frac{2\sqrt{R}}{1+R}\frac{1+\cos^3\theta_\mathrm{m}}{1-\cos\theta_\mathrm{m}}\right)S_\mathrm{r}^{-1} \tag{33}$$

for the maximum temperature error $\Delta T = T' - T$ due to photonic effects of a reflective surface. We see that the apparent temperature differences are minimized by minimizing the relative frequency difference between the emission lines $\Delta\omega/\overline{\omega}$ and the reflectivity R of the nearby reflective surface, and maximizing the NA (small $\cos\theta_\mathrm{m}$). For a Boltzmann thermometer, we can calculate the apparent temperature error analytically using the expression for the relative sensitivity $S_\mathrm{r} = \Delta E/k_\mathrm{B}T^2$

$$\Delta T \approx \left(\frac{k_\mathrm{B}}{\hbar\overline{\omega}}\frac{2\sqrt{R}}{1+R}\frac{1+\cos^3\theta_\mathrm{m}}{1-\cos\theta_\mathrm{m}}\right)T^2. \tag{34}$$

We observe the quadratic temperature dependence of the temperature error ΔT on the set temperature T, which explains the larger temperature error of the Boltzmann thermometer at elevated set temperatures (Figure 2g of the main text). Note that the maximum emission angle that can be collected by the microscope objective $\theta_\mathrm{m} = \arcsin(\mathrm{NA}/n)$ varies with the refractive index n at the position of the emitter due to refraction.

S5.4 Magnitude of photonic artifacts in Pinol *et al.*

We estimate that the maximum photonic errors in the experiments of Pinol et al.[S13] are $\Delta T_\mathrm{max} = 11$ K. To arrive at this estimate, we consider the effect of reflections on the interface between mitochondria and the cytoplasm. We use eq 4 of the main text with the following input parameters: the relative sensitivity of the thermometer is $S_\mathrm{r} = 1.2\%\ \mathrm{K}^{-1}$ at temperatures of 300–310 K (Figure S12b in Ref. S13), the relative frequency difference of the relevant emissions of Eu^{3+} and Sm^{3+} is $\Delta\omega/\overline{\omega} = 0.055$ (Figure 3c of Ref. S13), the reflectivity at the interface between mitochondria and cytoplasm is $R = (1.36 - 1.41)^2/(1.36 + 1.41)^2 = 0.00033$ (using refractive index data from Ref. [S14]). We estimate the maximum emission angle that can be collected by microscope objective in the experiments of Pinol et al.[S13] from the quoted resolution of $D = 1\ \mu$m (page 13 in the SI of Ref. [S13]) and the average wavelength $\lambda = 635$ nm of the relevant emissions: $\theta_\mathrm{m} = \arcsin[\lambda/(2Dn)] = 0.24$.

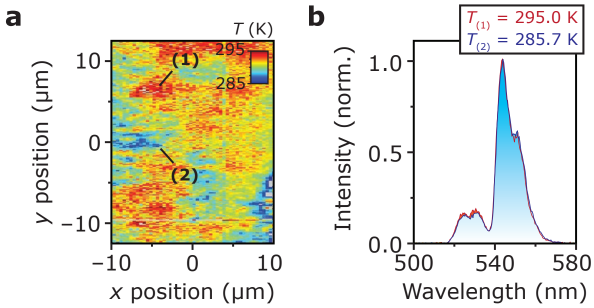

Figure S8 | Intensity-ratio distortions on a purely dielectric sample. (a) Temperature map of a purely dielectric sample indicating the hottest position (1) and the coldest position (2). **(b)** Corresponding emission spectra of the hottest position (1) (red, $T_{(1)} = 295.0$ K) and coldest position (2) (blue, $T_{(2)} = 285.7$ K). The differences are subtle but significant, and they correlate with the presence of polystyrene microspheres (see Figure 4 in the main text). Based on these emission spectra we find a maximum read-out error $\Delta T_{\mathrm{max}} = 9.3$ K .

Supporting References

(S1) Geitenbeek, R.G.; Prins, P.T.; Albrecht, W.; van Blaaderen, A.; Weckhuysen, B.M.; Meijerink, A. $NaYF_4$:Er^{3+},Yb^{3+}/SiO_2 core/shell upconverting nanocrystals for luminescence thermometry up to 900 K *J. Phys. Chem. C* **2017**, *121*, 3503–3510.

(S2) Rabouw, F.T.; Prins, P.T.; Villanueva-Delgado, P.; Castelijns, M.; Geitenbeek, R.G.; Meijerink, A. Quenching pathways in $NaYF_4$:Er^{3+},Yb^{3+} upconversion nanocrystals *ACS Nano* **2018**, *12*, 4812–4823.

(S3) Homann, C.; Krukewitt, L.; Frenzel, F.; Grauel, B.; Würth, C.; Resch-Genger, U.; Haase, M. $NaYF_4$:Yb,Er/$NaYF_4$ core/shell nanocrystals with high upconversion luminescence quantum yield *Angew. Chem. Int. Ed.* **2018**, *57*, 8765–8769.

(S4) van Swieten, T.P.; Yu, D.; Yu, T.; Vonk, S.J.W.; Suta, M.; Zhang, Q.; Meijerink, A.; Rabouw, F.T. A Ho^{3+}-based luminescent thermometer for sensitive sensing over a wide temperature range *Adv. Opt. Mater.* **2021**, *9*, 2001518.

(S5) Capobianco, J.A.; Kabro, P.; Ermeneux, F.S.; Moncorge, R.; Bettinelli, M.; Cavalli, E. Optical spectroscopy, fluorescence dynamics and crystal-field analysis of Er^{3+} in YVO_4 *Chem. Phys.* **1997**, *214*, 329–340.

(S6) Kunz, R. E.; Lukosz, W. Changes in fluorescence lifetimes induced by variable optical environments *Phys. Rev. B* **1980**, *21*, 4814–4828.

(S7) Dodson, C.M.; Zia, R. Magnetic dipole and electric quadrupole transitions in the trivalent lanthanide series: calculated emission rates and oscillator strengths *Phys. Rev. B* **2012**, *86*, 125102.

(S8) Karaveli, S.; Zia, R. Spectral tuning by selective enhancement of electric and magnetic dipole emission *Phys. Rev. Lett.* **2011**, *106*, 193004.

(S9) Sokolov, V.I.; Zvyagin, A.V.; Igumnov, S.M.; Molchanova, S.I.; Nazarov, M.M.; Nechaev, A.V.; Savelyev, A.G.; Tyutyunov, A.A.; Khaydukov, E.V.; Panchenko, V.Y. Determination of the refractive index of β-$NaYF_4$/Yb^{3+}/Er^{3+}/Tm^{3+} nanocrystals using spectroscopic refractometry *Opt. Spectrosk.* **2015**, *118*, 609–613.

(S10) DeFranzo, A.C.; Pazol, B.G. Index of refraction measurement on sapphire at low temperatures and visible wavelengths *Appl. Opt.* **1993**, *32*, 2224–2234.

(S11) Cocina, A.; Brechbühler, R.; Vonk, S.J.W.; Cui, J.; Rossinelli, A.A.; Rojo, H.; Rabouw, F.T.; Norris, D.J Nanophotonic approach to study excited-state dynamics in semiconductor nanocrystals *J. Phys. Chem. Lett.* **2022**, *13*, 4145–4151.

(S12) McPeak, K.; Jayanti, S.V.; Kress, S.J.P.; Meyer, S.; Iotti, S.; Rossinelli, A.A.; Norris, D.J. Plasmonic films Can easily be better: rules and recipes *ACS Photonics* **2015**, *2*, 326–333.

(S13) Pinol, R.; Zeler, J.; Brites, C.D.S; Gu, Y.; Téllez, P.; Carneiro Neto, A.N.; da Silva, T.E.; Moreno-Loshuertos, R.; Fernandez-Silva, P.; Gallego, A.I.; Martinez-Lostao, L.; Martínez, A.; Carlos, L.D.; Míllan, A. Real-time intracellular temperature imaging using lanthanide-bearing polymeric micelles *Nano Lett.* **2020**, *20*, 6466–6472.

(S14) Haseda, K.; Kanematsu, K.; Noguchi, K.; Saito, H.; Umeda, N.; Ohta, Y. Significant correlation between refractive index and activity of mitochondria: single mitochondrion study *Biomod. Opt. Express* **2015**, *6*, 859–869.
